# Using pERK immunostaining to quantify neuronal activity induced by stress in zebrafish larvae

**DOI:** 10.1016/j.xpro.2022.101731

**Published:** 2022-09-30

**Authors:** Laura Corradi, Margherita Zaupa, Suphansa Sawamiphak, Alessandro Filosa

**Affiliations:** 1 Max Delbrück Center for Molecular Medicine in the Helmholtz Association (MDC), Berlin, Germany; 2 Freie Universität Berlin, Institute for Biology, Berlin, Germany; 3 DZHK (German Center for Cardiovascular Research), Partner Site Berlin, Berlin, Germany

**Keywords:** Microscopy, Model organisms, Neuroscience, Behavior

## Abstract

The larval zebrafish has emerged as a very useful model organism to study the neuronal circuits controlling neuroendocrine and behavioral responses to stress. This protocol describes how to expose zebrafish larvae to hyperosmotic stress and test whether candidate populations of neurons are activated or inhibited by the stressor using a relatively rapid immunofluorescence staining approach. This approach takes advantage of the phosphorylation of the extracellular signal-regulated kinase (ERK) upon neuronal activation.

For complete details on the use and execution of this protocol, please refer to Corradi et al. (2022).

## Highlights

• Induction of stress in zebrafish larvae by using a hypertonic solution
• Immunofluorescence staining of pERK to quantify stressor-induced neuronal activity
• Steps to acquire confocal images and quantify pERK immunofluorescence intensity

## Abstract (editor highlights)

Publisher’s note: Undertaking any experimental protocol requires adherence to local institutional guidelines for laboratory safety and ethics.

## Graphical abstract

**Figure:**
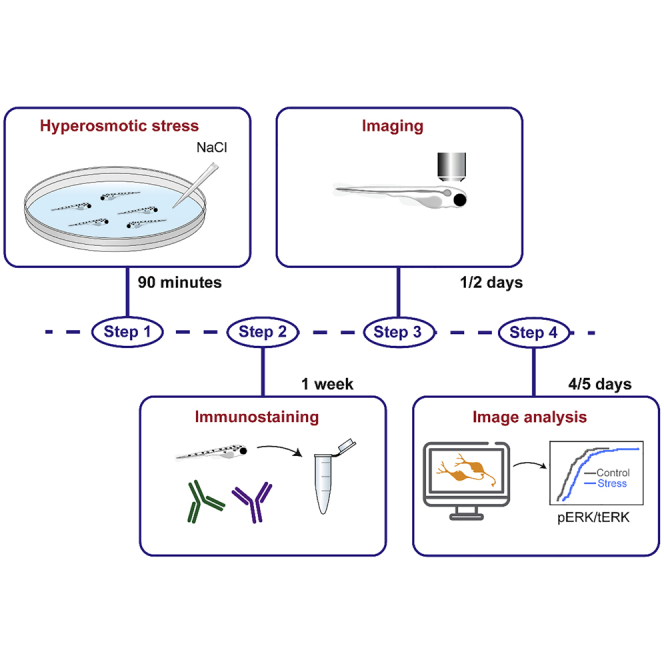

## Before you begin

This protocol describes the steps to expose larval zebrafish to hyperosmotic stress, and a detailed description of how to perform immunostaining of phosphorylated ERK (pERK). Finally, we provide information on how to acquire confocal images and to quantify fluorescence intensity. ERK is rapidly phosphorylated following neuronal activation (Rosen et al., 1994). pERK content in a neuron is a reliable readout of its activity in zebrafish and other vertebrates (Randlett et al., 2015). This protocol is specifically designed to verify, in a relatively fast way, if candidate neuronal types are responsive to stressors, before proceeding with further analysis of neuronal dynamics, using for example Ca^2+^ imaging methods. pERK staining can also be employed as a method for unbiased discovery of neurons involved in specific behaviors and sensory processing (Randlett et al., 2015).

Different sections of this protocol can also be used independently from the others, and in combination with other assays to study responses to stress in zebrafish. For example, the hyperosmotic stress section can be used for analyzing behavioral responses to stressors (Corradi et al., 2022; Ryu and De Marco, 2017), or to quantify cortisol content (Yeh et al., 2013). Moreover, the section describing immunofluorescence staining of pERK could be used as a quick method to quantify the activity of candidate populations of neurons in response to exposure to several types of sensory stimuli.

For this protocol, we used a transgenic line to label a specific group of neurons. However, neuronal types could also be identified by immunostaining or *in situ* hybridization of specific neuronal markers, used in combination with pERK immunostaining.

### Institutional permissions

All animal procedures described in this protocol were conducted in accordance with institutional (Max Delbrück Center for Molecular Medicine), State (LAGeSo Berlin), and German ethical and animal welfare guidelines and regulations.

### Fish husbandry

It is important to highlight that many different conditions can cause unwanted stress to zebrafish larvae and thus affect the analysis of neuronal activity. To ensure reproducibility, zebrafish larvae should be handled with extreme care, paying attention to all the critical steps included in this section.

***Note:*** Proper zebrafish husbandry is fundamental for ensuring animal welfare and avoiding stress. Adult zebrafish used for this protocol were kept in an animal facility with the following parameters: temperature = 26°C, pH = 7.4, water conductivity = 800 μS, 12:12 h light/dark cycle, maximum animal density = 5 adult fish/liter, diet: dry food two times a day and, after the 10^th^ day of development, also artemia once a day. Housing conditions with slightly different parameters should not affect the outcome of the experiments, as long as they are in line with the general housing recommendations suggested in (Aleström et al., 2020).

**CRITICAL:** We recommend using animals with the same genetic background in all the experimental groups as different laboratory strains display different stress susceptibility (Gorissen et al., 2015).

1. Place animals for breeding in the late afternoon in groups of 5–8 fish per tank. The size of the mating tanks used in this study was 1.7 L.

***Note:*** During breeding, the animals should be handled with care. Acute net handling stress was reported to increase the amount of whole body cortisol (Ramsay et al., 2009). This might affect the results of the experiments, as maternal stress may alter the development of the stress axis in the progeny (Nesan and Vijayan, 2016; Best et al., 2017).

2. The next morning, collect the fertilized eggs and transfer them into Petri dishes (60 mm diameter) filled with Danieau’s medium. Split the embryos into groups of 50 per Petri dish.

**CRITICAL:** Zebrafish embryos/larvae are raised with a maximum density of approximately 50 per Petri dish. A higher number of animals might cause crowding stress and influence the outcome of the experiments.

***Note:*** Be careful when choosing the Petri dish as several models used for cell culture contain at the bottom a microfilm which can be sticky for the embryos and larvae.

3. Keep the Petri dishes containing the zebrafish embryos in an incubator at 28.5°C on a 14 h/10 h light/dark cycle.

**CRITICAL:** We advise using incubators with programmable light/dark cycles. If the incubator does not have this feature, LED strip lights controlled by a timer can be installed. In zebrafish, as in mammals, stress-regulating circuits and the production of cortisol are influenced by circadian clues, including light exposure (Yeh, 2015). Therefore, alteration of light/dark exposure might affect the outcome of the experiments.

**CRITICAL:** Zebrafish embryos and larvae should not be kept for long periods of time at different temperatures or different light conditions than the ones indicated above. If a different raising procedure is required for the experiment, make sure that all the experimental groups are exposed to the same conditions and for the same periods of time.

***Note:*** Although 1-phenyl 2-thiourea (PTU) is commonly used to avoid pigmentation in zebrafish larvae, we advise avoiding its use for investigating neuronal activity, since it alters behavior and nervous system function (Antinucci and Hindges, 2016). To obtain transparency, you can perform a *post mortem* procedure to clear the tissue prior to immunostaining, as described in step 8 of the step-by-step method details section. Alternatively, homozygous *mitfa* mutants lacking melanophores (Lister et al., 1999) can be used.

4. Replace the Danieau’s solution every two days to ensure that the embryos/larvae are raised in a fresh medium.
5. Sort the zebrafish larvae using a fluorescence stereo microscope once the expression pattern of transgenic markers is visible.

***Note:*** This protocol was established using clutchmate larvae, which require sorting from the same petri dish, and therefore similar exposure time to fluorescent light. If some of the experimental groups do not need to be checked for expression, we advise to expose them approximately to the same conditions as you would do for the other groups, i.e., time at room temperature, exposure to dark and/or fluorescent light, etc., since these parameters can influence stress (Ryu and De Marco, 2017).

**CRITICAL:** The fluorescence sorting should not be done on the same day of the experiment as this is a source of stress for the fish. We advise conducting it at the latest 24 h prior to the experiment.

6. Keep the zebrafish larvae in the incubator until the desired developmental stage is reached, which for this protocol is 5 days post fertilization (dpf).

***Note:*** If the experiment requires the use of older animals, feeding and other parameters that could affect responses to stress should be taken into account.

## Key resources table

**Table:** 

REAGENT or RESOURCE	SOURCE	IDENTIFIER
**Antibodies**		

polyclonal chick Anti-GFP. Dilution 1:500	Thermo Fisher Scientific	Cat# A10262; RRID: AB_2534023
monoclonal mouse Anti-p44/42 MAP Kinase (tERK). Dilution 1:500	Cell Signaling	Cat# 4696; RRID: AB_390780
monoclonal rabbit Anti-Phospho p44/42 MAP Kinase (Thr202/Tyr204) (pERK). Dilution 1:500	Cell Signaling	Cat# 4370: RRID: AB_2315112
Alexa Fluor 488 Anti-chicken. Dilution 1:300	Thermo Fisher Scientific	Cat# A11039; RRID: AB_142924
Alexa Fluor 647 Anti-mouse. Dilution 1:300	Cell Signaling	Cat# 4410S; RRID: AB_1904023
Alexa Fluor 555 Anti-rabbit. Dilution 1:300	Cell Signaling	Cat# 4413; RRID: AB_10694110

**Chemicals, peptides, and recombinant proteins**		

Trypsin-EDTA	Sigma	T4049
Agarose, Low Melting Point (mounting medium)	Roboklon	E0303-50
Tricaine (3-amino benzoic acidethylester)	PHARMAQ	n/a
Dimethyl sulfoxide (DMSO)	Th. Geyer	23419.3
Triton X 100	Roth	3051.3
Bovine Serum Albumin (BSA)	SERVA	11943.02
Paraformaldehyde (PFA) 16%	Thermo Fisher Scientific	28908
Phosphate-buffered saline (PBS)	Sigma	P4417-100TAB
Potassium hydroxide (KOH)	Alfa Aesar	A16199
Methanol (100%, HPLC grade)	Carl Roth	4627.1
Sodium Chloride (NaCl)	SERVA	39781.02
Tris	Sigma	T1503-1KG
Hydrochloric acid (HCl)	Sigma	H1758-500ml
Hydrogen peroxide (H_2_O_2_) 30%	ChemCruz	sc-203336A
(4-(2-hydroxyethyl)-1-piperazineethanesulfonic acid) (HEPES)	Roth	9105.4
Potassium chloride (KCl)	ChemCruz	sc-203207
Magnesium sulfate (MgSO_4_)	ChemCruz	sc-211764
Calcium nitrate (Ca(NO_3_)_2_)	Honeywell/Fluka	C1396-500G
Goat serum	Sigma	Cat# G6767

**Experimental models: Organisms/strains**		

Zebrafish larvae 5 dpf: *TgBAC[galn:GAL4-VP16,myl7:mCherry]*^*mpn213*^. Undefined sex	Förster et al. (2017)	ZFIN: ZDB-ALT-170908-13
Zebrafish larvae 5 dpf: *Tg[5xUAS:EGFP]^nkuasgfp1a^*. Undefined sex	Asakawa et al. (2008)	ZFIN: ZDB-ALT-080528-1

**Software and algorithms**		

Fiji/ImageJ	Schindelin et al. (2012)	https://imagej.nih.gov/ij/
ZEN Black software	ZEISS	N/A

**Other**		

Fluorescence Stereo Microscope	Olympus	SZX16
Zeiss LSM880 Confocal Microscope	ZEISS	N/A
Water-immersion Objective (W Plan- Apochromat 20×/1.0 DIC VIS-IR)	ZEISS	421452-9800-000
Thermoblock (ThermoMixer C)	Eppendorf	5382000015
Petri dish (35 mm)	Sarstedt	82.1135.500
Petri dish (60 mm)	Sarstedt	83.3901
Plastic pipette 3 mL	Pastette	LW4111
pH-Meter	METTLER TOLEDO	Five Easy
Serological pipettes	INTEGRA	Pipet Boy Acu 2

## Materials and equipment

**Table:** 1× PBS

Reagent	Final concentration	Amount
PBS	1×	5 tablets
MilliQ H_2_O	N/A	Add to 1 L
**Total**	N/A	**1 L**

***Note:*** Mix until the tablets are dissolved. Autoclave and store at room temperature (∼23°C) for long-term storage (up to six months).

**Table:** 1× PBT

Reagent	Final concentration	Amount
1× PBS	1×	Add to 500 mL
Triton X 100	0.3%	1.5 mL
**Total**	N/A	**500 mL**

***Note:*** Mix and stir until dissolved and store at room temperature. As Triton is viscous, we advise using a scale or a specific pipette designed for viscous liquids. Store at room temperature (∼23°C) for long-term storage (up to six months).

**Table:** 4% PFA in PBT

Reagent	Final concentration	Amount
**1× PBT**	1×	30 mL
**PFA 16%**	4%	10 mL
Total	N/A	**40 mL**

***Note:*** Dissolve 1 glass ampoule (10 mL) of PFA in PBT. Make 1 mL aliquots and store them at −20°C. Thaw 4% PFA solution at room temperature before using it. Thawed 4% PFA can be stored at 4°C for up to one week.

**CRITICAL:** PFA is a hazardous chemical and it should be prepared under a chemical hood and using personal protective equipment.

**Table:** 30× Danieau’s medium

Reagent	Final concentration	Amount
NaCl	1,740 mM	101.7 g
KCl	21 mM	1.56 g
MgSO_4_	12 mM	2.96 g
Ca(NO_3_)_2_	18 mM	4.25 g
HEPES	150 mM	35.75 g
MilliQ H_2_O	N/A	bring up to 1 L
**Total**	N/A	**1 L**

***Note:*** Mix and adjust the pH to 7.6. Autoclave and store at room temperature (∼23°C) for long-term storage (up to six months).

**Table:** 1× Danieau’s medium

Reagent	Final concentration	Amount
30× Danieau’s medium	1×	300 mL
MilliQ H_2_O	N/A	8,700 mL
**Total**	N/A	**9,000 mL**

***Note:*** Store at room temperature (∼23°C) for long-term storage (up to six months).

**Table:** 200 mM NaCl

Reagent	Final concentration	Amount
NaCl	200 mM	2.33 g
Danieau’s medium	1×	Bring up to 200 mL
**Total**	N/A	**200 mL**

***Note:*** Mix and stir until dissolved. Store at room temperature (∼23°C) for long-term storage (up to three months). Discard the solution if salt precipitates form.

**Table:** 10% KOH

Reagent	Final concentration	Amount
KOH	10%	10 g
MilliQ H_2_O	N/A	Bring up to 100 mL
**Total**	N/A	**100 mL**

***Note:*** Mix and stir until the crystals dissolve, and store at room temperature (∼23°C) for long-term storage (up to six months).

**CRITICAL:** KOH is corrosive and should be handled with care and using personal protective equipment.

**Table:** Clearing solution

Reagent	Final concentration	Amount
H_2_O_2_ 30%	3%	100 μL
KOH 10%	0.5%	50 μL
PBT	1×	850 μL
**Total**	N/A	**1 mL**

***Note:*** The solution should be prepared fresh immediately before use.

**CRITICAL:** H_2_O_2_ and KOH can cause irritation to the skin and eyes and should be handled with care using personal protective equipment. Be careful as once the solution is in contact with the samples the clearing reaction will cause formation of bubbles.

**Table:** Blocking solution

Reagent	Final concentration	Amount
Goat serum	5%	500 μL
BSA	1%	0.1 g
DMSO	1%	100 μL
PBT	1×	Bring up to 10 mL
**Total**	N/A	**10 mL**

***Note:*** Weight the BSA directly in a 15 mL Falcon tube. Thaw an aliquot of goat serum and add it, together with the remaining reagents, to the falcon tube containing the BSA. Vortex vigorously and store the blocking solution at 4°C for up to one week.

**Table:** 1.5% agarose

Reagent	Final concentration	Amount
Agarose, low melting point	1.5%	1.5 g
1× PBS	1×	Bring up to 100 mL
**Total**	N/A	**100 mL**

***Note:*** Add the agarose to the PBS and heat it up in the microwave until the agarose is completely dissolved. Aliquot it and store it at 4°C for up to three months. Before usage, warm the aliquot at 70°C for 10 min. Transfer the aliquot 30 min prior to use at 50°C and keep it at 50°C while mounting to avoid solidification.

**Table:** Tricaine

Reagent	Final concentration	Amount
Tricaine	4 mg/mL	2 g
1× Danieau’s medium	1×	Bring up to 500 mL
**Total**	N/A	**500 mL**

***Note:*** Dissolve the tricaine into Danieau’s solution using a magnetic stirrer. Adjust the pH to 7.5 and store aliquots at −20°C for up to one month. Once thawed, store the tricaine solution at 4°C for up to 24 h.

## Step-by-step method details

### Hyperosmotic stress

**Timing: 90 min**

This section describes how to expose freely swimming zebrafish larvae to hyperosmotic stress prior to immunostaining (described in this protocol) or other types of staining, such as HCR *in situ* hybridization (Choi et al., 2018). The protocol described here was optimized for 5 dpf zebrafish larvae.

***Note:*** Administration of a hypertonic solution is an established stressor for zebrafish larvae. In this protocol, we treated zebrafish larvae with NaCl at a final concentration of 100 mM. Higher or lower concentrations of NaCl might also be used (Yeh et al., 2013; De Marco et al., 2014).

**CRITICAL:** We advise to be consistent with the timing of the experiments as production of the neuropeptide corticotropin-releasing hormone (crh), which is responsible for the activation of the stress axis, was shown to fluctuate during the day (Yeh, 2015). Therefore, it is likely that neurons modulating stress are affected. For this protocol the experiments were performed in the early afternoon.

1. Approximately 75 min prior to the experiment, transfer 20 to 25 larvae to a new Petri dish (60 mm diameter) using a 3 mL plastic pipette.
  a. Gently tilt the Petri dish and remove as much Danieau’s medium as possible.
  b. Rapidly fill the Petri dish with 13 mL of fresh Danieau’s medium.

***Note:*** We add 13 mL instead of 15 mL of medium as approximately 2 mL will remain in the tilted Petri dish during this procedure.

2. Leave the Petri dish containing the zebrafish larvae at room temperature on the bench for 60 min under light conditions before the experiment.

***Note:*** In this way, the animals will have enough time to recover from potential stress received during the transfer procedure.

**CRITICAL:** Prior to the experiment the larvae should not be disturbed. If possible, place the Petri dishes on a vibration-free table as even touching the bench might cause vibrations in the water, which could stress the fish.

3. Using a serological pipette add 15 mL of Danieau’s solution to one Petri dish (control group) and 15 mL of 200 mM NaCl in Danieau’s medium to the other Petri dish (stressed group).

***Note:*** In this way, each petri dish will contain a final volume of 30 mL. We distribute the solution with the pipette evenly across the Petri dish, and do not swirl the dish.

4. After 10 min add 1.3 mL of tricaine to each Petri dish and rapidly transfer the larvae to two different 1.5 mL microcentrifuge tubes.
5. Remove all the solution inside the microcentrifuge tubes and replace it with 1 mL of 4% PFA/PBT.

***Note:*** You can spin down the microcentrifuge tubes and use a glass Pasteur to rapidly remove the solution and avoid that the larvae stick to the plastic walls of the pipette.

**CRITICAL:** PFA is a hazardous chemical and should be always handled under a chemical hood and using personal protective equipment.

**CRITICAL:** This step must be performed rapidly as tricaine causes a decrease in neuronal activity (Turrini et al., 2017). No more than 90 s should pass from the administration of tricaine to the addition of PFA.

### Immunostaining

This section describes how to perform immunofluorescence staining to detect pERK and total ERK (tERK).

#### Preparation of the samples

**Timing: 60 min without step 8, 120 min with step 8, plus overnight fixation (12–16 h)**

***Note:*** To perform the washes we advise using a 3 mL plastic pipette with a 10 μL tip inserted on the top of it to avoid losing the larvae. The 1.5 mL microcentrifuge tubes can be inserted in a 50 mL Falcon tube placed on a rotator.

6. Fix the larvae for ∼18 h at 4°C placing the 1.5 mL microcentrifuge tubes horizontally without agitation to ensure that all the larvae are homogeneously covered with PFA.

**CRITICAL:** Do not add more than 25 larvae in one 1.5 mL microcentrifuge tube as this might result in an improper fixation of the samples.

***Note:*** Alternatively, larvae can be fixed for one hour at room temperature. However, in our experience, this leads to higher variability in the fixation of the larvae and penetration of the antibodies.

7. Remove the PFA and wash the larvae three times with 1 mL of 1× PBT for 15 min at room temperature under agitation.

**CRITICAL:** PFA is a hazardous chemical and should be handled using personal protective equipment. Discard the liquid waste containing the PFA under a chemical hood and dispose of it according to the safety instructions of your workplace.

8. (Optional) Clear the samples to remove melanin pigments:
  a. Incubate the larvae with 500 μL of a solution containing 3% H_2_O_2_ + 0.5% KOH in PBT.
  b. Seal the lid of the 1.5 mL tubes with parafilm and place them in a thermoblock at room temperature (∼23°C) with 300 rpm agitation for 15 min.
  c. Carefully remove the clearing solution trying to remove all the bubbles that will form.
  d. Rinse the larvae with 1 mL of 1× PBS.
  e. Wash the larvae under agitation three times with 1 mL of 1× PBT for 15 min.

***Note:*** The clearing solution should be prepared fresh immediately before use. H_2_O_2_ is unstable and should be kept in a dark container and discarded after six months from the opening.

**CRITICAL:** H_2_O_2_ can cause skin and eye irritation. Handle the solution with care and wear protective equipment. Once you open the lid of the 1.5 mL microcentrifuge tube after the incubation be careful as there will be a large amount of bubbles.

**Pause point:** The samples can be stored at 4°C for up to four days before proceeding with the next steps. Alternatively, they can be stored at −20°C for longer periods of time in MeOH (see steps 9 and 10).

#### Dehydration of the samples with MeOH for long storage (optional)

**Timing: 2 days (40 min the first day, and 30 min the day after)**

This step is advisable whenever the samples are going to be stored for longer periods than one week before proceeding with the next steps of the protocol. Consider that treatment with MeOH generally leads to the disappearance of endogenous fluorescent signals.

***Note:*** To perform the washes we advise using a 3 mL plastic pipette with a 10 μL tip inserted on the top of it to avoid losing the larvae. The 1.5 mL microcentrifuge tubes can be inserted in a 50 mL Falcon tube placed on a rotator.

9. Dehydrate the larvae with a series of washes (1 mL each) under agitation at room temperature:
  a. 25% MeOH / 75% PBT for 5 min.
  b. 50% MeOH / 50% PBT for 5 min.
  c. 75% MeOH / 25% PBT for 5 min.
  d. 100% MeOH for 15 min.
  e. Replace with fresh 100% MeOH and store the larvae at −20°C.

**Pause point:** Larvae can be stored at −20°C for long periods of time. In our experience up to one year.

10. Rehydrate the larvae with a series of washes (1 mL each) under agitation:
  a. 75% MeOH / 25% PBT for 5 min.
  b. 50% MeOH / 50% PBT for 5 min.
  c. 25% MeOH / 75% PBT for 5 min.
  d. 100% PBT for 5 min (repeat three times).

#### Permeabilization of the samples

**Timing: 80 min**

This part describes how to permeabilize and block the samples for immunostaining. As with the rest of the protocol, these steps are optimized for 5 dpf zebrafish larvae. Other methods of permeabilization will require different concentrations and incubation times, and their efficiency with the desired antibodies should be tested beforehand.

***Note:*** To perform the washes we advise using a 3 mL plastic pipette with a 10 μL tip inserted on the top of it to avoid losing the larvae. The 1.5 mL microcentrifuge tubes can be inserted in a 50 mL Falcon tube placed on a rotator.

11. Incubate the larvae in 500 μL of 150 mM Tris-HCl in PBT for 5 min at room temperature without agitation.
12. Transfer the tubes containing the larvae with the 150 mM Tris-HCl solution to a thermoblock set at 70°C and incubate without agitation for 15 min.
13. Remove the solution and wash the larvae under agitation three times with 1 mL of 1× PBT for 15 min.
14. Incubate the larvae in Trypsin-EDTA (diluted 1:50 in PBT) for 40 min on ice without agitation.

**CRITICAL:** The concentration of the permeabilization solution and incubation time indicated here are optimized for 5 dpf larvae. Younger or older embryos/larvae will require different concentrations and incubation times.

***Note:*** This incubation period can be used to prepare the blocking solution (see materials and equipment section) that will be needed for step 16.

15. Remove the solution and wash the larvae under agitation three times with 1 mL of 1× PBT for 15 min.

**Pause point:** If needed, the larvae can be left in PBT at 4°C for up to 16 h.

#### Blocking and incubation with antibodies

**Timing: 75 min + 4 days (incubation with primary antibodies), 60 min washes + 2 days (incubation with secondary antibodies)**

This section describes how to prepare the samples for immunostaining with antibodies against the following proteins: GFP, pERK, and tERK (see key resources table). Incubation times and concentration of the antibodies may be different if different antibodies than the ones used for this protocol are employed.

***Note:*** To perform the washes we advise using a 3 mL plastic pipette with a 10 μL tip inserted on the top of it to avoid losing the larvae. The 1.5 mL microcentrifuge tubes can be inserted in a 50 mL Falcon tube placed on a rotator.

***Note:*** We used a transgenic line labeling with GFP hypothalamic neurons producing the neuropeptide galanin (*TgBAC[galn:GAL4-VP16,myl7:mCherry]*^*mpn213*^*; Tg[5xUAS:EGFP]^nkuasgfp1a^*). Hence the use of an anti-GFP antibody.

***Note:*** The use of an antibody against tERK is necessary to normalize the amount of pERK for the amount of total (phosphorylated and not) ERK in the neurons during the image analysis process.

16. Incubate the larvae in 700 μL of blocking solution for one hour at room temperature under agitation.

***Alternatives:*** The larvae can also be incubated overnight (12–16 h) at 4°C under agitation. In our experience, there is no difference in the outcome of the results but it is always advisable to be consistent with different experiments.

***Note:*** 700 μL is the minimum volume required to ensure that the larvae in the 1.5 mL microcentrifuge tubes are homogeneously covered by the solution. If smaller or larger tubes are used, adjust the volume accordingly.

17. Prepare the antibody solution diluting the antibodies in 1,400 μL of blocking solution (for two experimental groups in two separate tubes). In this protocol the following antibodies were used:

**Table:** Primary antibody solution (for two groups of larvae)

Reagent	Dilution	Amount
polyclonal chick Anti-GFP	1:500	2.8 μL
monoclonal mouse Anti-p44/42 MAP Kinase (tERK)	1:500	2.8 μL
monoclonal rabbit Anti-phospho p44/42 MAP Kinase (Thr202/Tyr204) (pERK)	1:500	2.8 μL
Blocking solution	N/A	Bring to 1,400 μL
**Total**	N/A	**1,400 μL**

**CRITICAL:** We recommend testing the efficacy of new antibodies and optimizing concentration and incubation time beforehand in a pilot assay. Optimization might be required also when using larvae at different developmental stages.

18. Remove the blocking solution and replace it with the primary antibody solution (700 μL for each tube) and incubate the larvae for 96 h at 4°C with agitation.

***Note:*** The antibody solution should be prepared shortly before incubation and according to the number of samples. We recommend not to prepare different antibody solutions for different samples as it might affect the outcome of the analysis.

19. Remove the primary antibody solution and wash the larvae under agitation at least three times with 1 mL of 1× PBT for 15 min at room temperature.

***Note:*** In our experience increasing the number of washes to remove excess of primary antibody decreases substantially the background.

20. Prepare the secondary antibody solution diluting the antibodies in 1,400 μL of blocking solution (for two tubes). In this protocol the following antibodies were used:

**Table:** Secondary antibody solution (for two groups of larvae)

Reagent	Dilution	Amount
Alexa Fluor 488 Anti-chicken	1:300	4.7 μL
Alexa Fluor 647 Anti-mouse	1:300	4.7 μL
Alexa Fluor 555 Anti-rabbit	1:300	4.7 μL
Blocking solution	N/A	Bring to 1,400 μL
**Total**	N/A	**1,400 μL**

21. Remove the PBT and replace it with the secondary antibody solution (700 μL for each tube) and incubate the larvae for 48 h at 4°C under agitation.

**CRITICAL:** Secondary antibodies should be protected from exposure to light. Cover the 1.5 mL microcentrifuge tubes with aluminum foil and work quickly during the preparation of the secondary antibody solution and the following washing steps to minimize light exposure.

22. Remove the secondary antibody solution and wash the larvae under agitation at least three times with 1 mL of 1× PBT for 15 min.

***Note:*** In our experience increasing the number of washes to remove the excess of secondary antibody decreases substantially the background. However, prolonged exposure to light during washes might cause fluorophore photobleaching.

23. Confirm, using at least one larva, that the antibody signal is detectable using a fluorescence microscope.

**Pause point:** Larvae can be stored in the dark at 4°C for up to three days. However, we advise proceeding with the imaging as soon as possible.

***Note:*** This protocol for pERK/tERK immunostaining is optimized for 5 dpf larvae. However, this method was previously used to quantify neuronal activity in younger and older fish, for example from as early as 3 dpf to juvenile stages (Fore et al., 2020).

### Imaging

This section describes how to image the immunostained samples, including the critical steps to obtain fluorescent images that can be reliably quantified.

***Note:*** The preparation of the samples and the imaging protocol might be adapted according to different types of confocal microscopes and/or objectives. We used an upright Zeiss LSM880 microscope equipped with a water-immersion objective (W Plan-Apochromat 20×/1.0 DIC VIS-IR).

#### Mounting

**Timing: ∼2 min per larva**

This section describes how to mount the zebrafish larvae in low-melting-point agarose for acquiring images using a confocal microscope.

**Figure 1:**
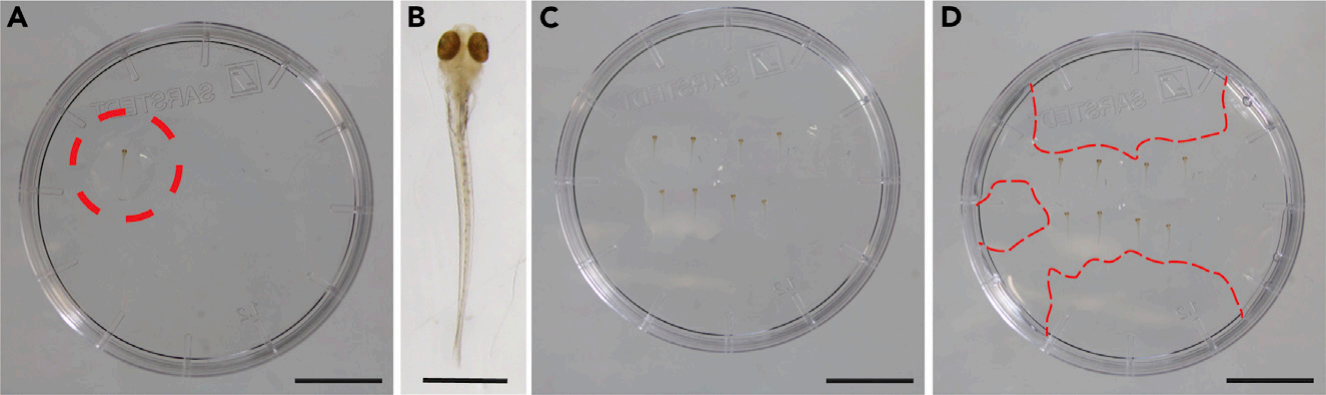
Sequence of images showing the process for placing larvae in agarose for microscopy (A and B) Initially a single larva is embedded in a drop of low-melting-point agarose on the lid of a Petri dish (A, dashed circle, and B at higher magnification). (C and D) Subsequently, the other larvae are added (C), and the solidified agarose in the center is connected to the border of the lid by adding more agarose with a pipette (D, the dashed red lines mark the borders of the agarose). Scale bars: 1 cm in (A, C, D); 1 mm in (B). See also Methods video S1.

24. Keep 1.5% low-melting-point agarose at 50°C while mounting to avoid solidification.
25. Mount the samples on the lid of a 35 mm plastic Petri dish (Figure 1 and Methods video S1).
  a. Using a plastic pipette transfer a larva to the Petri dish’s lid and remove as much PBT as possible.
  b. Using a glass Pasteur pipette add one drop of agarose on top of the larva.
  c. Using a 10 μL pipette tip, gently orient the larva with its dorsal part up to expose the brain for imaging.
  d. Repeat the process for all the larvae you intend to image alternating samples belonging to different treatment conditions.
  e. Once the agarose surrounding the larvae is solidified, add agarose to connect the wall of the Petri dish’s lid to the larvae to avoid the sample to move during imaging.

**CRITICAL:** Orient the larvae as quickly as possible as the agarose solidifies after approximately two minutes at room temperature. Avoid touching the head while mounting to avoid disrupting the tissue. In addition, add as little agarose as possible to embed the larvae to ensure that the brain is within the working distance of the microscope objective.

**CRITICAL:** While mounting, always alternate larvae belonging to different treatment groups to avoid exposing one group to light for more time than the other. Different exposure times to light of different experimental groups could lead to detection of fluorescence intensity differences during the analysis caused by photobleaching of the fluorophores rather than by the treatment itself.

26. Once the agarose is solidified, cover the Petri dish’s lid containing the samples with PBS (if you will use a water immersion objective) and proceed with the imaging.

***Note:*** Mounting medium and orientation of the larvae must be changed according to the objective and the microscope that you intend to use.

Methods video S1. Procedure for embedding zebrafish larvae in agarose, related to step 25 The video displays the main steps for placing the zebrafish larvae in agarose to acquire images at the confocal microscope.

#### Setup of parameters for confocal imaging

**Timing: ∼30 min, varies depending on the number of samples**

This section describes the main steps for acquiring confocal images to perform quantitative fluorescence intensity analysis. The settings described here apply to the Zen Black software and an upright Zeiss LSM 880 confocal microscope.

**CRITICAL:** This protocol does not substitute a training for the use of a confocal microscope that should be given by experienced personnel. Use safety measures to avoid laser-induced eye damage.

**Figure 2:**
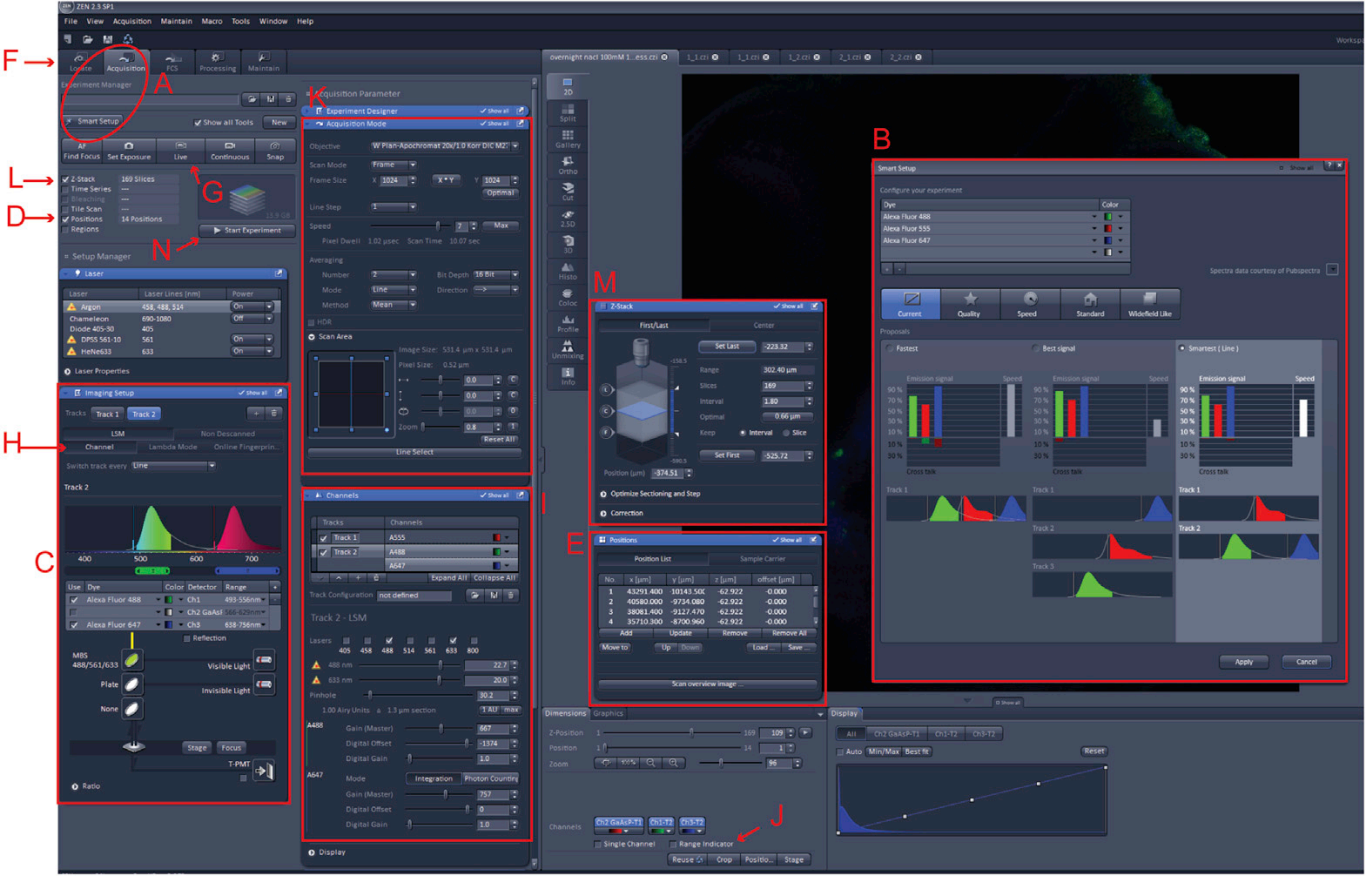
Image showing the main panel of the Zeiss Zen Black software The red letters and rectangles indicate the steps required to set up the imaging session.

***Note:*** We advise choosing the setting “Smartest” as in our experience it is the best compromise between imaging speed and cross-channels signal contamination.

27. Switch on the computer, microscope, and fluorescent lamp according to the instructions provided by Zeiss.
28. Launch the microscope software (Zen Black) and switch on the lasers you intend to use as they might need some minutes to warm up.
29. Place the Petri dish’s lid containing the larvae on the microscope stage.
30. Press “Acquisition” on the top left of the Zen Black main window, and then select “Smart Setup” to choose laser, light, path, and filters in an automatic way (Figure 2A).
  a. In the “Smart Setup” window (Figure 2B) select the dyes and the pseudocolors you intend to use. For example, Alexa-fluor 488 and green; Alexa-fluor 555 and red; Alexa-fluor 647 and blue.
  b. Chose the acquisition mode “Smartest” from the three options: “Fastest'', ''Best Signal'', and ''Smartest'' (Figure 2B).

31. On the left, under “Imaging setup” (Figure 2C) double-check that the detection range and the laser chosen by the software for each fluorophore are correct.
32. Click on “Positions” (Figure 2D) and move the “Position” window at the center of the screen (Figure 2E). Press the “Locate” tab on the top left corner (Figure 2F) and open the shutter of the fluorescent lamp.
  a. Locate the first mounted zebrafish larva and focus on the brain.
  b. In the “Position” window press “Add” (Figure 2E).
  c. Repeat steps a and b for all the samples.
  d. Save the position list (Figure 2E).
  e. Using the function “Move to” (Figure 2E) you are now able to automatically move the microscope stage to each larva.

***Note:*** We highly recommend saving the positions of all the samples in order to spend less time for the adjustment of the imaging settings and for the imaging itself. This step can also be performed while waiting for the lasers to warm up.

***Note:*** Remember to do this step and the next ones always after using the function “Smart Setup” (step 30) otherwise the settings will be lost.

33. Press again the “Acquisition” tab to continue with the setting of the imaging parameters.
34. Press “Live” (Figure 2G) and focus on the area of interest and adjust the settings by pressing “Channels” (Figure 2H).
  a. For each laser set a “Laser Power” (Figure 2I). The laser power should be enough to observe the fluorescent signal but set as low as possible to reduce photobleaching.
  b. Set “Gain (Master)” (Figure 2I) while using the ‘Range Indicator’’ (Figure 2J) to visualize saturation (Figure 3).

**Figure 3:**
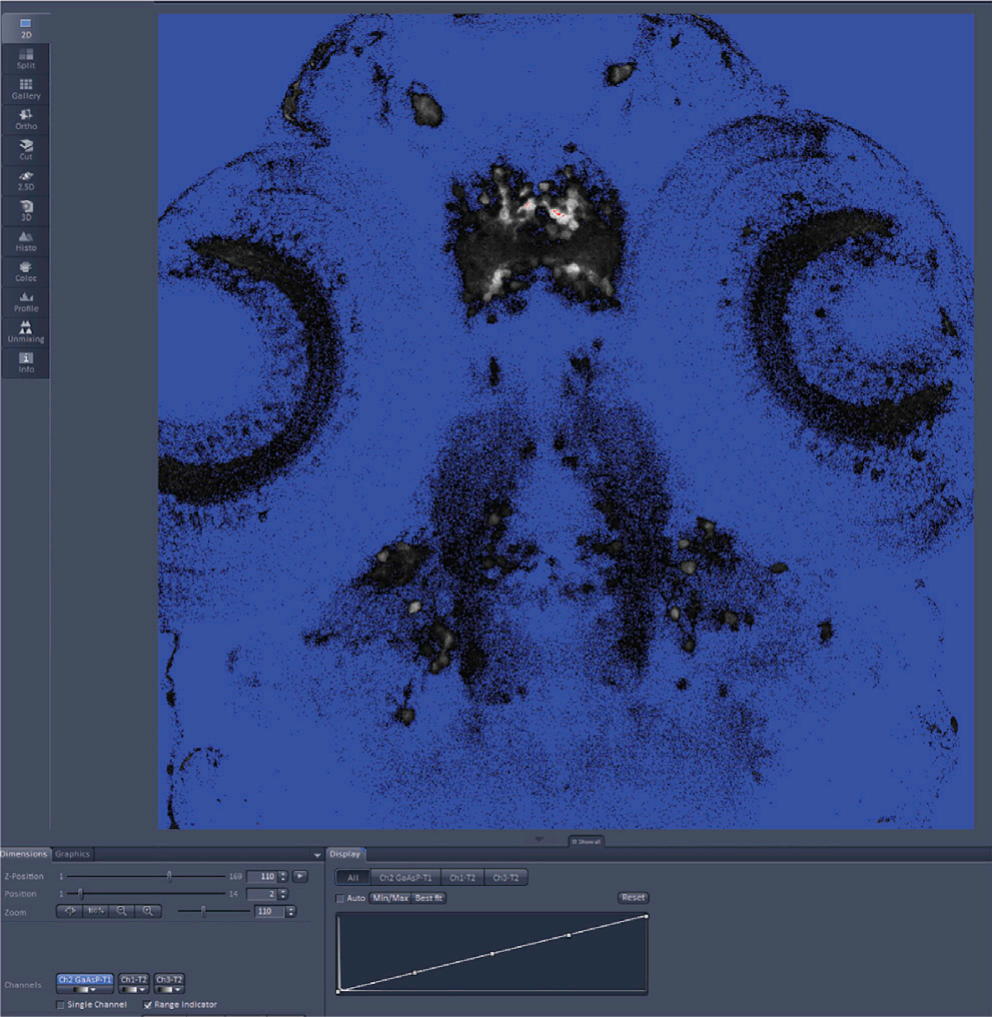
Picture of the image preview window in the Zeiss Zen Black software when the “Range Indicator” option is selected Pixels with intensities below the detection threshold are labeled in blue, and saturated ones in red.
  c. Do not change “Digital Offset” and “Digital Gain” (Figure 2I).
  d. Set the “Pinhole Diameter” at 1 AU (Airy Unit) (Figure 2I).
  e. Repeat all the previous steps for each channel.
35. Once you have chosen the settings for each channel, move the stage using the “Move to” function in the “Position” window (Figure 2E).
  a. Verify that the fluorescence signal is below saturation in each fish (Figure 3).
  b. If in the region of interest in any of the larvae the fluorescence signal is saturated, lower the “Gain” and/or the “Laser power”.

**CRITICAL:** It is fundamental that the fluorescence signal in the area of interest is below saturation in all the samples. A saturated signal will compromise the final analysis.

#### Z-stack acquisition

**Timing: ∼5/10 min per larva depending on the thickness of the region of interest**

This section describes how to set the Zen Black software to acquire confocal Z-stack multichannel images. It includes the description of the settings to enable automated imaging of several larvae in the same Petri dish.

***Note:*** Using a different software will most likely require different settings.

36. Go to the “Acquisition Mode” window (Figure 2K) to select the type of acquisition, image resolution, and scanning speed.
  a. Chose “Frame” in the “Scan Mode” menu.
  b. Select 1024 × 1024 in the “Frame Size”. In our experience, this resolution is a good compromise between time required for imaging and image quality.
  c. Set the “Speed”. We advise choosing a minimum of 8 as a high scan speed reduces the time required for image capture.
37. In the “Acquisition Mode” window (Figure 2K) chose “Averaging” to acquire multiple scans for noise reduction.
  a. Chose the “Number” of scans that will be acquired for each plane. In our experience, two scans are sufficient to reduce significantly the noise.
  b. Select under “Method” whether the pixels of the multiple scans will be summed (“Sum“) or averaged (“Mean”). We advise to choose the “Mean” option.
  c. Under “Bit Depth’’ select 16. It is recommended to choose a high bit depth for performing quantification of fluorescent signals.
  d. Chose the “Direction” of the scan. We advise choosing “Unidirectional” as “Bidirectional” is faster but not ideal when acquiring multi-track images.
38. Move the stage to the first larva to be imaged and select the option “Z-stack” (Figure 2L).
  a. Focus on the top border of your region of interest, and in the “Z-stack“ window click “Set Last” (Figure 2M).
  b. Move the focus until you reach the bottom of your region of interest and click “Set first”.
  c. Click “Optimal” to set automatically the slice number based on the parameters you selected. Alternatively, the parameters can be set manually.
  d. Click the center of the Z-stack “C” on the left.
  e. Go to the “Position” window (Figure 2E) and select “Update”.
  f. Repeat steps a-e for each sample.

***Note:*** We advise not to use an interval between slices larger than 1.5 μm.

***Alternatives:*** It is possible to acquire the Z-stack images of all the larvae automatically. In this case, you will have to make sure that the Z-stack is thick enough to encompass the region of interest in every sample. Using this automatic function, the Z-stack parameters will be the same for all the stage positions. In this case, proceed as follow:

39. (Optional) Set the automatic imaging of all the larvae.
  a. In the “Position” window use “Move to” to move to each larva you desire to image (Figure 2E).
  b. Check that your region of interest is within the range of the Z-stack and press the center of the Z-stack clicking “C” on the left (Figure 2M).
  c. Go to the “Position” window and select “Update”.
  d. Repeat the procedure for each larva.
40. On the top left of the main window press “Start Experiment” (Figure 2N) to acquire the Z-stack(s).
41. Save your images by pressing “Save as” choosing the extension .czi. or .tif.

### Image analysis

This section describes the steps to quantify fluorescence intensity of the acquired images using ImageJ/Fiji.

#### Measurement of fluorescence intensity

**Timing: ∼5 min per cell**

This section describes how to select the regions of interest (ROIs) using ImageJ/Fiji and measure fluorescence intensities of the pERK and tERK channels.

**Figure 4:**
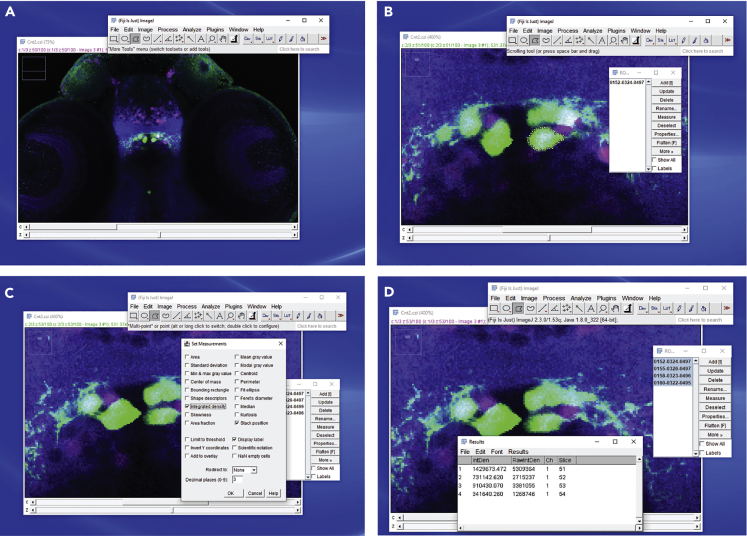
Steps required to quantify fluorescence signals in ImageJ/Fiji (A) Example of a group of genetically-labeled neurons stained with anti-GFP (green), anti-pERK (magenta), and anti-tERK (blue) antibodies. The main control panel of Fiji is also shown. (B) Zoomed-in image showing one of the neurons of interest (in green) marked by a ROI, and the Fiji ROI window. (C) Images showing the “Set measurements” window. (D) Images showing the “Results” window.

***Note:*** We previously used this method to quantify the activity of a genetically-labeled (with GFP) population of neurons in the hypothalamus (Corradi et al., 2022). Alternatively, the desired neuronal types could be identified using fluorescence *in situ* hybridization or immunostaining of specific neuronal markers.

42. Open a multichannel image with ImageJ/Fiji and zoom on the neurons you intend to analyze (Figures 4A and 4B).

43. Click on “Analyze”→ “Tools” → “ROI manager” to open the ROI window (Figure 4B).
44. Select the first neuron you intend to analyze.
45. For each neuron set the ROIs for the measurement of the fluorescence intensity (Figure 4B).
  a. Move the stack to the most ventral plane where your neuron of interest is visible.
  b. Carefully draw the ROI over the outline of the neuron using the “Polygon selections” tool. The channel with the labeling of the marker used to identify the neuronal population of interest (in this example GFP) should be used for this step.
  c. Click “Add” in the ROI window.
  d. Repeat the operation for each plane in which the neuron is visible.
  e. Save the ROIs by clicking “More”→ “Save” in the ROI manager window.
46. Measure the fluorescence intensity of each channel in the selected ROIs (Figures 4C and 4D).
  a. Click “Analyze” → “Set measurements” and select “Integrated density”.
  b. Select one of the channels of the image you want to measure, for example pERK.
  c. Select all the ROIs labeling your neuron of interest in the ROI window.
  d. Click “Measure” in the ROI window.
  e. Select another channel of your image, for example tERK.
  f. Repeat steps c and d.
47. Repeat steps 45 and 46 for all the neurons of interest.
48. After measuring the fluorescence intensity of pERK and tERK immunostaining you can copy-paste the measurements into your favorite data-processing software, such as Excel, Python, R, etc.

#### Analysis of the pERK/tERK ratios

**Timing: ∼2 min per neuron**

This section describes how to calculate the pERK/tERK ratios, which will provide a readout of the activity of the neurons of interest in the different treatment groups.

49. Use your favorite data-processing software (Excel, Python, R, etc.) to calculate for each neuron the total fluorescence intensities of each channel.
  a. Sum the pERK Integrated Densities values of each plane of your neuron of interest.
  b. Repeat the operation for every neuron.
  c. Repeat steps a and b for the tERK channel.
50. For each neuron calculate the ratio between the sums of pERK and tERK Integrated Densities values.
51. Plot your data and perform statistical analyses to compare pERK/tERK values between the stressed and not-stressed groups.

***Note:*** We usually plot pERK/tERK values in the form of cumulative distributions and determine statistical significance using a two-sample Kolmogorov-Smirnov test in Python. However, you should select the type of data-representation method and statistical analysis most suitable to your experimental design. Variance of the data will likely vary depending on the specific type of neuronal population under study and on the specific stimuli utilized, and will affect the number of neurons and animal required for the analysis.

## Expected outcomes

Following the steps described in this protocol you will be able to verify whether candidate populations of neurons in the brain of zebrafish larvae are activated or inhibited by exposure to hypertonic stress, by comparing pERK/tERK ratios from neurons in stressed and control, non-stressed, fish. Some practical examples of the application of this protocol can be found in our recent publication (Corradi et al., 2022).

## Limitations

This protocol is designed to obtain a relative estimate, compared to unstressed controls, of the activity of candidate neuronal populations in larval zebrafish exposed to hyperosmotic stress using immunofluorescence staining of pERK. This method is ideal for testing in a relatively rapid way, especially if you plan to check many different candidate neuronal populations, whether specific neuronal types are responsive to a stressor (or eventually to other sensory stimuli). However, pERK content is an indirect way to measure neuronal activity and does not allow to obtain precise information about the activation dynamics of neurons. For this reason, once one or more populations of neurons are identified with this method, we strongly suggest to use *in vivo* techniques for measuring neuronal activity, such as Ca^2+^ imaging, to confirm the results and investigate in more details the dynamics of neuronal responses.

Furthermore, this method allows detection of differences of average activity of a population of neurons, potentially hiding differences of activation patterns in subpopulations. For example, in a recent study in which we used this protocol, we detected on average increased activity in a hypothalamic population of neurons following exposure to hypertonic stress (Corradi et al., 2022). Subsequent Ca^2+^ imaging analysis confirmed that the majority of neurons were indeed activated by the stressor. However, we also found that a minority of the neurons were instead inhibited by or unresponsive to the stimulus (Corradi et al., 2022).

## Troubleshooting

### Problem 1

Fish appear unhealthy or die after application of the hyperosmotic solution.

### Potential solution

In our experience, zebrafish larvae tolerate well the hyperosmotic stress protocol. If larvae die during or immediately after the exposure to the NaCl solution (see step 3), it is most likely due to the fish’s starting health conditions. Before proceeding with the protocol always ensure that by 4 dpf the larvae display an inflated swim bladder and normal spontaneous locomotion*.*

### Problem 2

No differences in neuronal activity are detected, or there is high inter-fish variability.

### Potential solution

Inability to detect differences in neuronal activation (see step 51) likely means that the population of neurons under study is not responsive to the applied stressor. However, it is highly advisable to include a positive control to rule out the possibility of technical problems. For example, Crh^+^ and Galn^+^ neurons in the preoptic area display increased pERK/tERK values after exposure to hyperosmotic stress (Corradi et al., 2022).

### Problem 3

Signal from one or more antibodies is not detected or is very weak.

### Potential solution

• Make sure to select the correct secondary antibodies, binding to the primary antibody from the right species (see steps 17 and 20). In addition, check that the isotypes are compatible.
• Do not over-fix the samples, which leads to poor antibody penetration. This problem can occur when performing the fixation at room temperature for longer than 1 h or at 4°C for longer than 18 h (see step 6).
• There might be a problem with penetration of the antibodies, especially if the signal from more than one antibody is weak. Ensure that the Trypsin solution is always kept on ice and that it is not expired. If the developmental stage is different from 5 dpf, ensure proper optimization of the incubation time in the Trypsin solution and of its concentration (see step 14). Alternatively, you can try different types of permeabilization methods, for example using Proteinase K.
• The incubation time might be too short. You can try to increase it, but the timing used in this protocol should be more than sufficient for larvae from 5 to 7 dpf. Immunostaining of younger or older larvae may require shorter or longer incubation times, respectively. You should perform pilot experiments testing several incubation times in case younger or older larvae are required for your experiment.
• Check that the antibodies have been stored correctly. If this is not the case you might need to use a new vial. Remember that fluorophores-conjugated secondary antibodies are light sensitive and should always be stored protected from light (see steps 20–22).
• The fluorophores might be bleached due to exposure to light or laser stimulation. Always protect your sample from light after incubation with secondary antibodies, and set the laser power as low as possible (see step 34a).
• Check that the microscope settings (i.e., excitation and emission parameters) are correct (see step 30).

### Problem 4

Nonspecific staining with one or more antibodies.

### Potential solution

• The antibodies might bind to other proteins in addition to the intended target. Test other antibodies against the same target. We suggest to use antibodies previously tested in zebrafish whenever possible.
• The concentration of the antibodies might be too high. Try reducing the amount of antibodies added to the samples (see steps 17 and 20).

### Problem 5

High background.

### Potential solution

• Increase the washing time and the number of washes after incubations with primary and secondary antibodies (steps 19 and 22).
• Blocking is a fundamental step for reducing the background. You can try to increase the time of incubation in the blocking solution up to 2 h at room temperature (step 16) and increase up to 7% the concentration of serum.
• Treatment with MeOH prior to immunostaining often helps with background issues. Try to perform steps 9 and 10, incubating for at least one night the samples at −20°C.
• During the washing steps, the larvae should never dry out as this might also be a source of high background signal.
• Sometimes background might be caused by autofluorescence of the sample. You can use a negative control not stained with the antibodies to check whether autofluorescence is the culprit, and adjust the confocal settings accordingly.

### Problem 6

Spectral crossover of fluorophore signals during imaging.

### Potential solution

• Always ensure to use fluorophores having as little overlap of emission and excitation spectra as possible. You can use several online tools to check the spectra of the fluorophores and choose your secondary antibodies accordingly. For example: www.thermofisher.com/order/fluorescence-spectraviewer.
• Try to narrow the detection range of the fluorophores in the confocal settings under “Range”. Alternatively, if overlap between fluorophores spectra is unavoidable, you can acquire different channels sequentially by choosing ''Best Signal'' in the “Smart Setup” menu (see step 30b).

## Resource availability

### Lead contact

Further information and requests for resources and reagents should be directed to and will be fulfilled by the lead contact, Alessandro Filosa (Alessandro.filosa@mdc-berlin.de).

### Materials availability

This study did not generate any new reagent.

## Data Availability

This study did not generate new data or code.
